# Supplementary material for: Host adaptive mutations in the 2009 H1N1 pandemic influenza A virus PA gene regulate translation efficiency of viral mRNAs via GRSF1
Source: Commun Biol. 2022 Oct 17;5:1102. doi: 10.1038/s42003-022-04082-5 (PMC9576711; doi:10.1038/s42003-022-04082-5)

a)

Virus	Amino Acid at PB2 residue										
	53	66	87	195	293	299	344	354	391	453	731
Cal	Arg	Met	Asn	Asp	Arg	Arg	Val	Ile	Glu	Ser	Val
Mich	Lys	Ile	Asp	Asn	Lys	Lys	Met	Leu	Asp	Thr	Ile

b)

Virus	Amino acid at PA residue:					
	100	224	321	330	362	438
Cal	Val	Pro	Asn	Ile	Arg	Ile
Mich	Ile	Ser	Lys	Val	Lys	Val

c)

Virus	Amino acid at PB1 residue:		
	154	397	435
Cal	Gly	Ile	Ile
Mich	Asp	Met	Thr

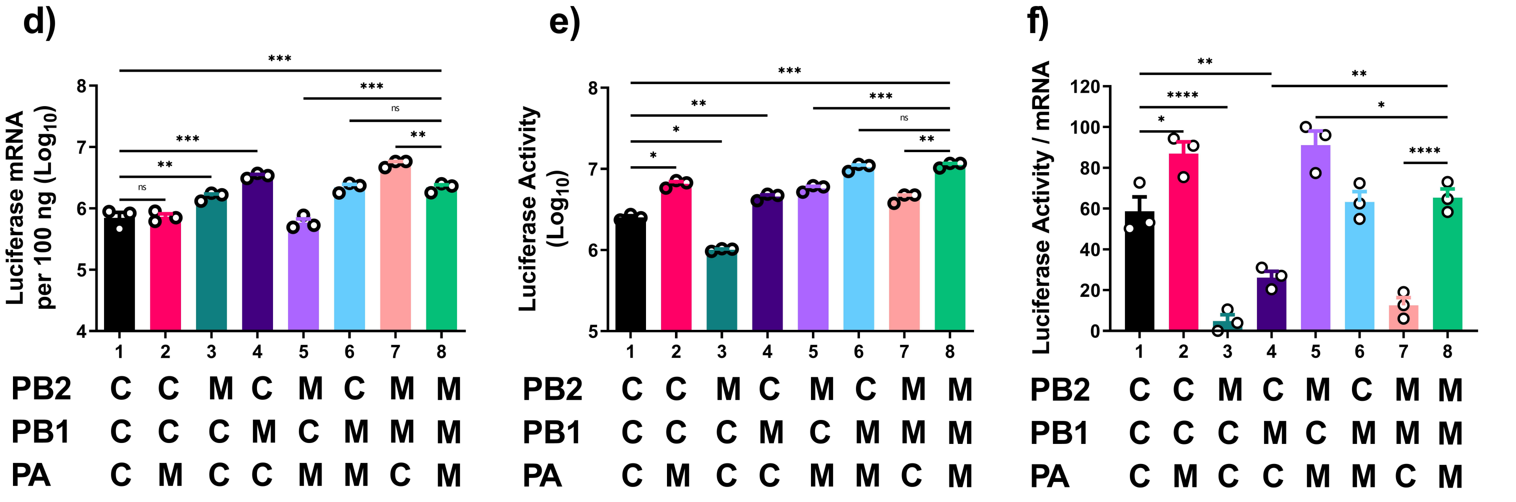

Supplementary Figure 1. Additional polymerase mutations introduced during seasonal circulation of pH1N1 further enhance translation efficiency. a Amino acid differences between A/California/04/2009(H1N1) "Cal" and A/Michigan/272/2017(H1N1) "Mich" PB2. **b** Amino acid differences between Cal and Mich PA **c** Amino acid differences between Cal and Mich PB1. **d-e** 293T cells were transfected with the indicated vRpRp subunits (C = Cal, M = Mich) along with Cal NP and pPolI-NP-luc. **d** Luciferase mRNA produced by vRdRp was quantified by qRT-PCR. **e** Luciferase activity determined by the Dual-Luciferase Reporter Assay system. **f** Ratio of luciferase activity per mRNA, transformed to a normal distribution. Error bars show means plus/minus the standard deviations (n = 3 biological replicates). One-way ANOVA followed by Tukey's multiple comparison test (* $P < 0.05$, ** $P < 0.01$, *** $P < 0.001$).

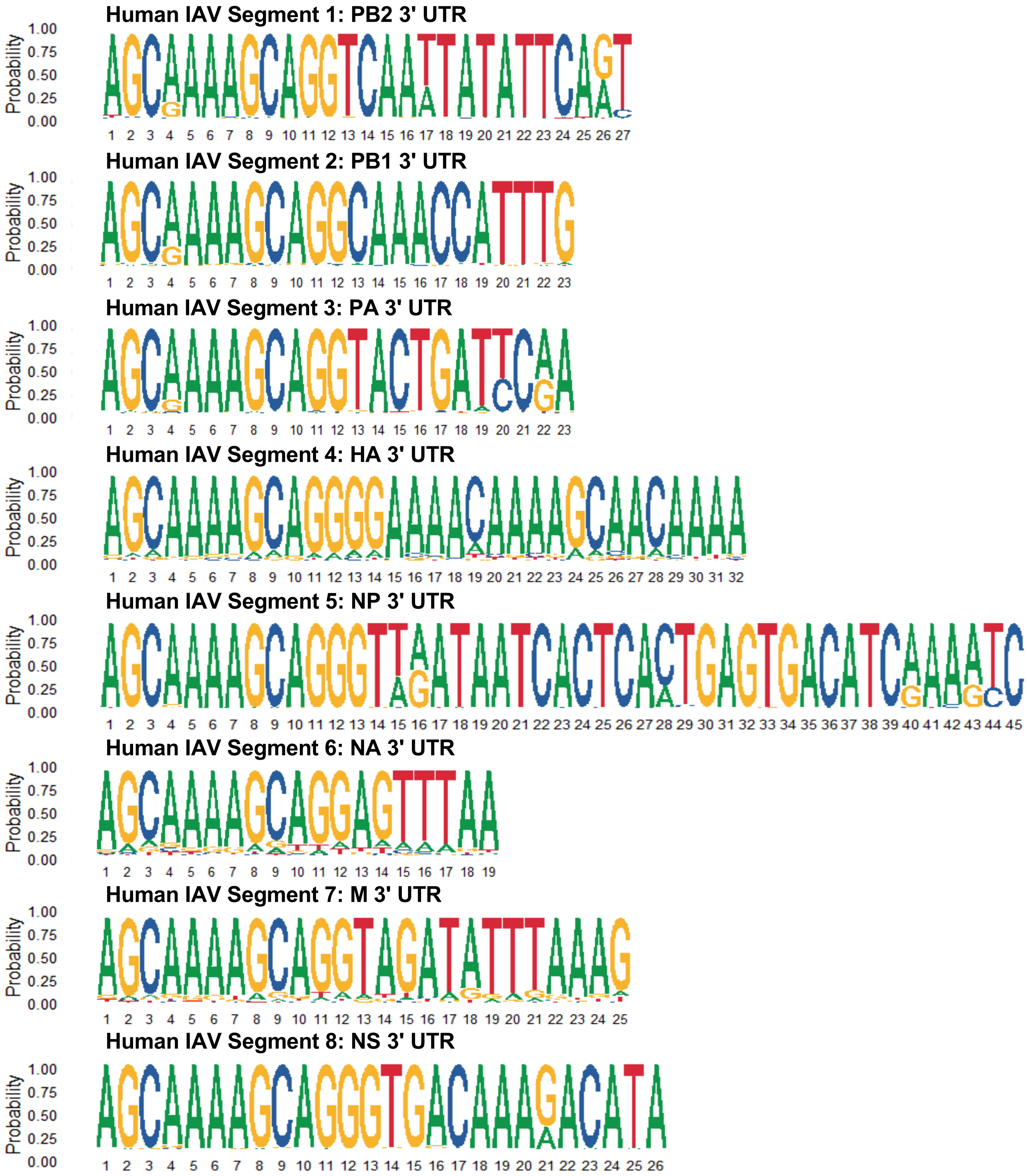

Supplementary Figure 2. Sequence comparison of human IAV 3' UTRs. Logo analysis for the 3' UTR for segments 1-8 in human IAVs. Analysis includes 1,133 full length (2341 bp) PB2 sequences, 1,297 full length (2339 bp) PB1 sequences, 1,168 full length (2233 bp) PA sequences, 1,017 full length (1760 bp) HA sequences, 1,338 full length (1565 bp) NP sequences, 795 full length (1440 bp) NA sequences, 502 full length (1011 bp) M sequences and 1,462 full length (890 bp) sequences. Sequences were obtained from fludb.org.

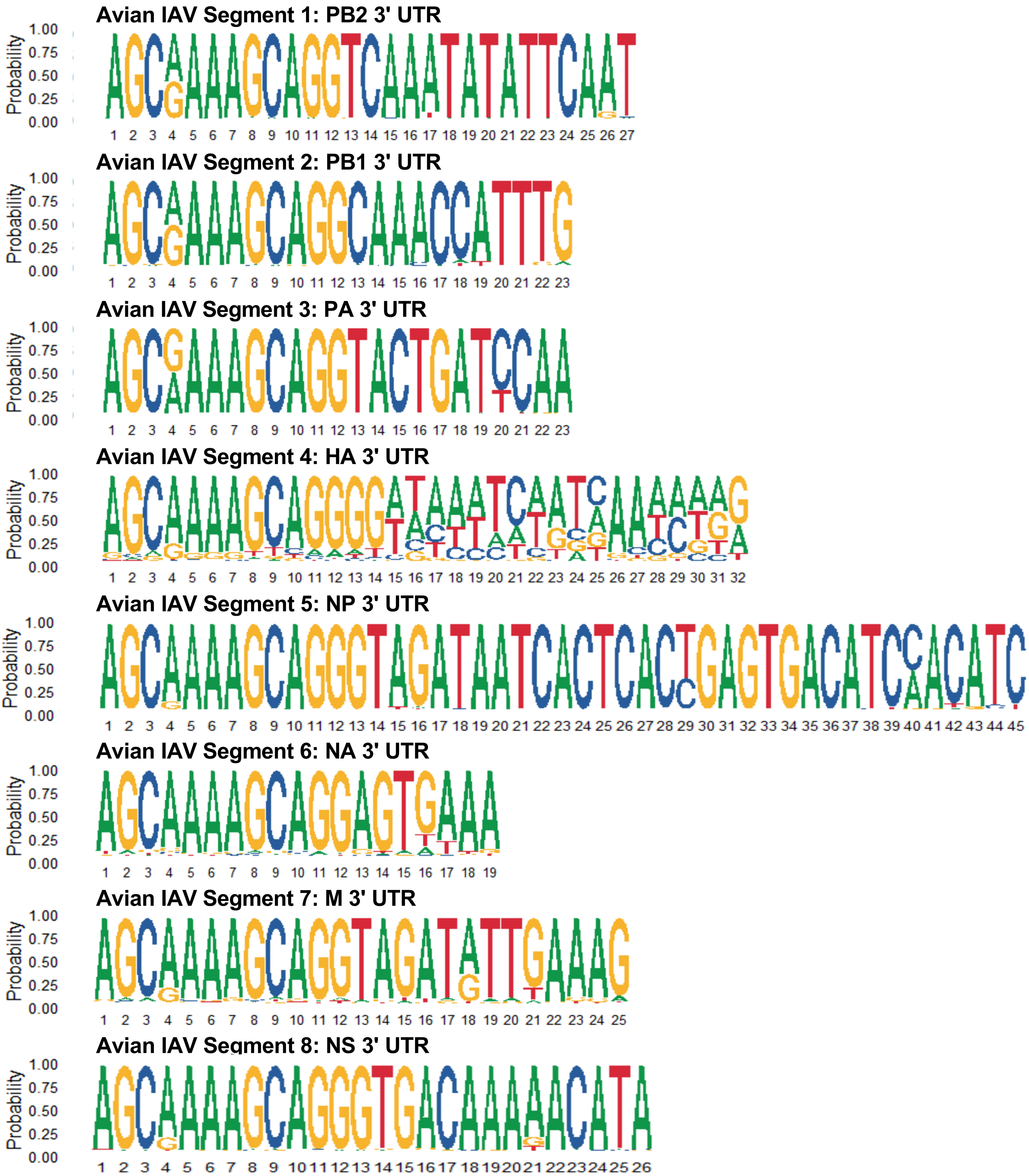

Supplementary Figure 3. Sequence comparison of chicken IAV 3' UTRs. Logo analysis for the 3' UTR segments 1-8 in chicken IAVs. Analysis includes 3,141 full length (2341 bp) PB2 sequences, 2,033 full length (2339 bp) PB1 sequences, 2,134 full length (2233 bp) PA sequences, 994 full length (1760 bp) HA sequences, 2,360 full length (1565 bp) NP sequences, 394 full length (1440 bp) NA sequences, 2,654 full length (1011 bp) M sequences and 2,125full length (890 bp) sequences. Sequences were obtained from fludb.org.

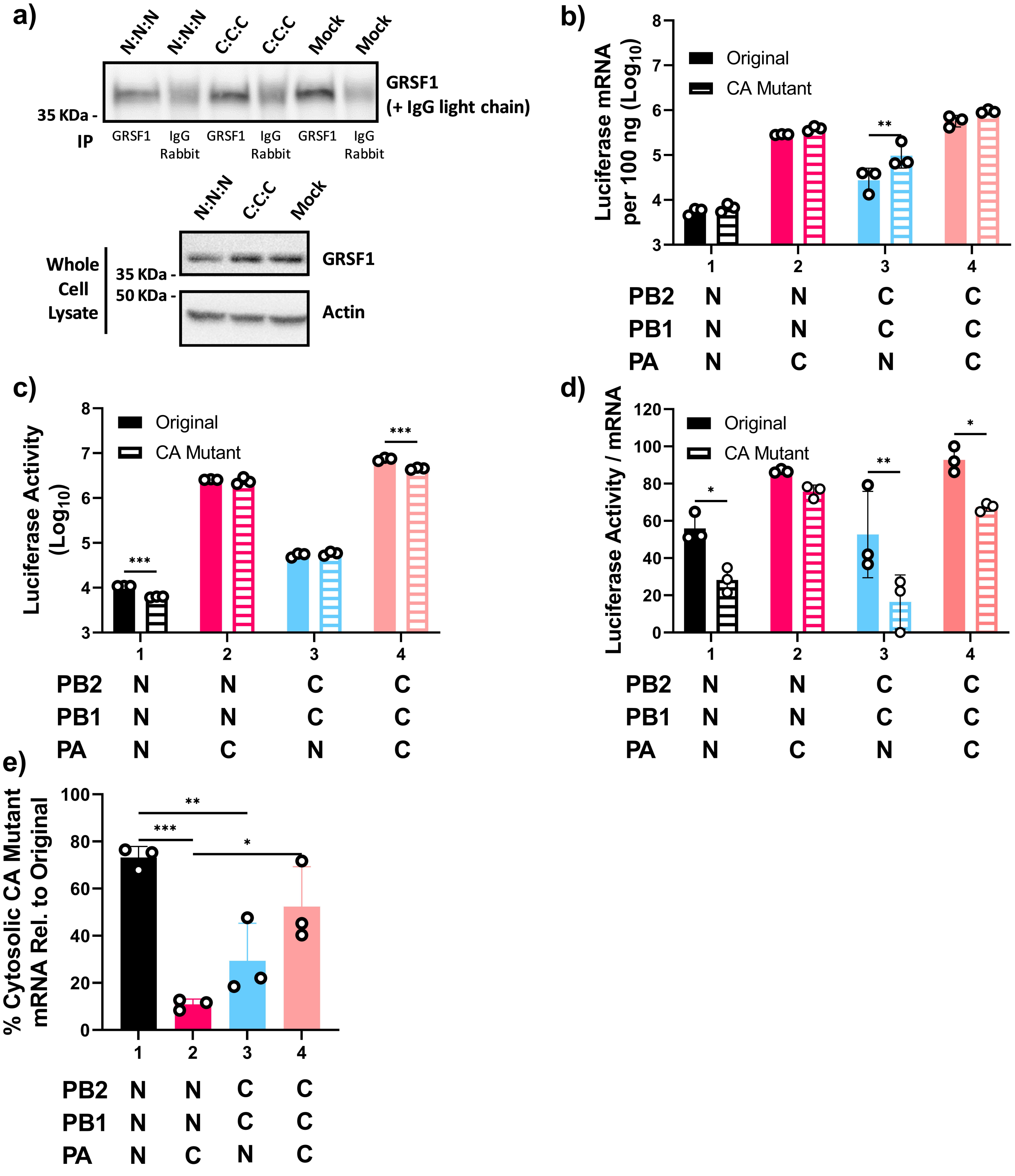

Supplementary Figure 4. GRSF1 binding regulates cytosolic mRNA levels and translation efficiency. a 293T cells were transfected with Nan or Cal vRdRp along with Cal NP and pPolI-NP-luc. Anti-GRSF1 rabbit or IgG rabbit control antibody were used for immunoprecipitation from whole cell lysates and the eluted materials were immunoblotted for GRSF1. The light chain from the IgG rabbit control runs at the same size as GRSF1. Whole cell lysates were also immunoblotted for GRSF1 and β-actin. **b-e** 293T cells were transfected with the indicated vRdRp subunits (N = Nan, C = Cal) along with Cal NP and pPolI-NP-luc. **b** Luciferase mRNAs from the original and mutated (CA) pPolI-NP-luc templates were quantified by qRT-PCR. **c** Luciferase activity was determined by the Dual-Luciferase Reporter Assay system. **d** Ratio of luciferase activity per mRNA, transformed to a normal distribution. **e** Percentages of cytosolic luciferase mRNAs produced from CA mutant relative to original are shown. All error bars show means plus/minus the standard deviations (n = 3 biological replicates). One-way or Two-way ANOVA followed by Tukey's multiple comparison test (* $P < 0.05$, ** $P < 0.01$, *** $P < 0.001$).

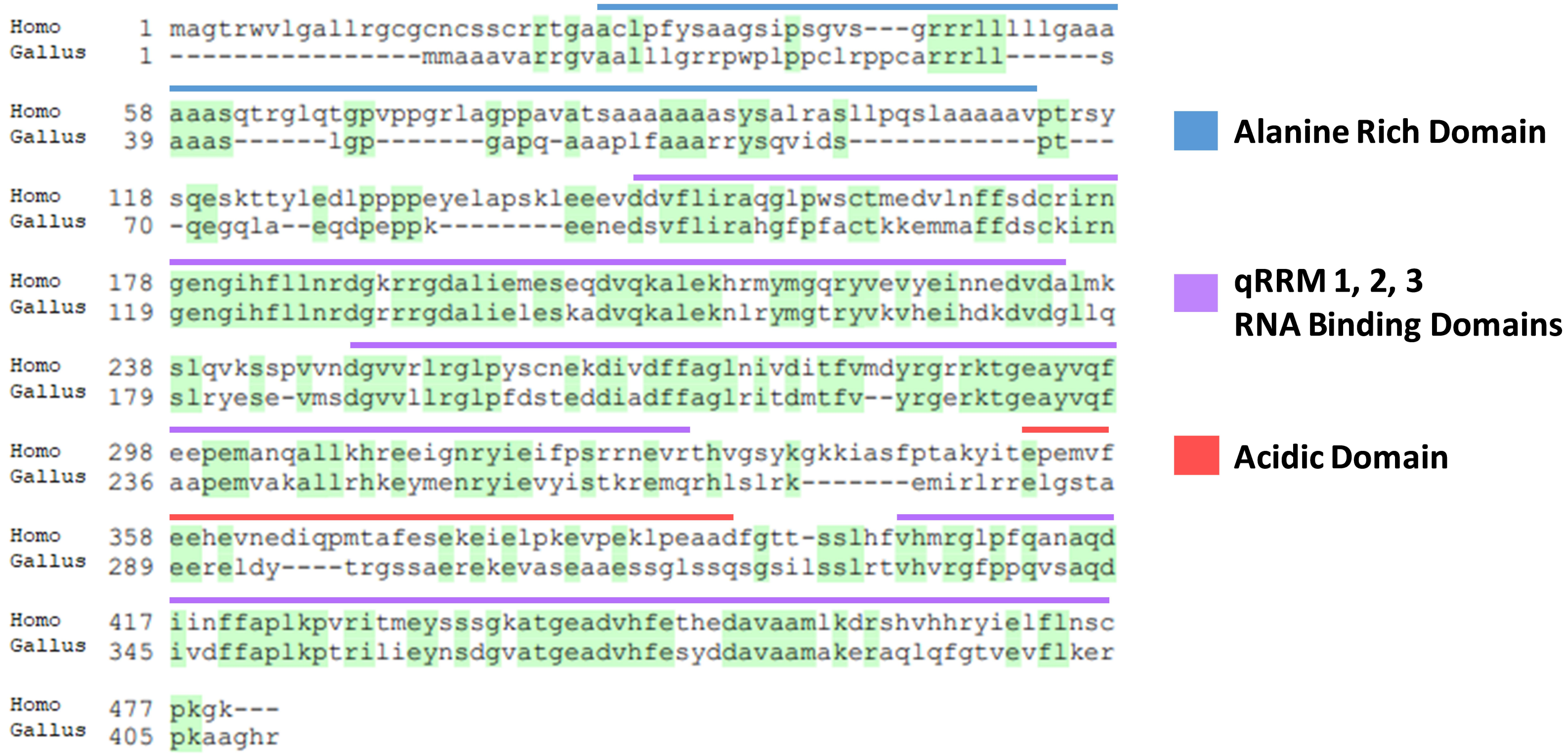

Supplementary Figure 5. Sequence comparison of human and chicken GRSF1. Sequence alignment of GRSF1 from *Homo sapiens* and *Gallus gallus* (NCBI Reference Sequence: NP_002083.4 and XP_015131904.2). Location of key domains in human GRSF1 is highlighted.

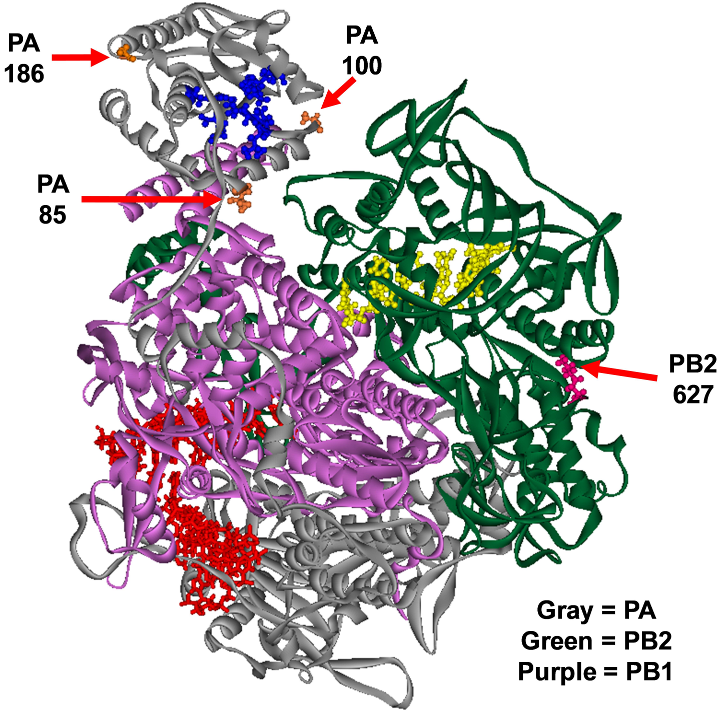

Supplementary Figure 6. Locations of PA residues 85, 100, and 186. Crystal structure of human H3N2 IAV polymerase (PDB: 6RR7). The PA, PB2, and PB1 subunits are shown in gray, green, and purple, respectively. An m7G capped RNA primer is highlighted in yellow in the cap-binding domain of PB2. A 5’-3’ vRNA promoter is highlighted in red in the PB1 active site. The endonuclease domain active residues of PA are highlighted in blue. The location of host adaptive PA mutations in the endonuclease domain are highlighted. The location of PB2 residue 627 is shown for reference.

Supplemental Figure 7: Uncropped versions of blots

Figure 1d

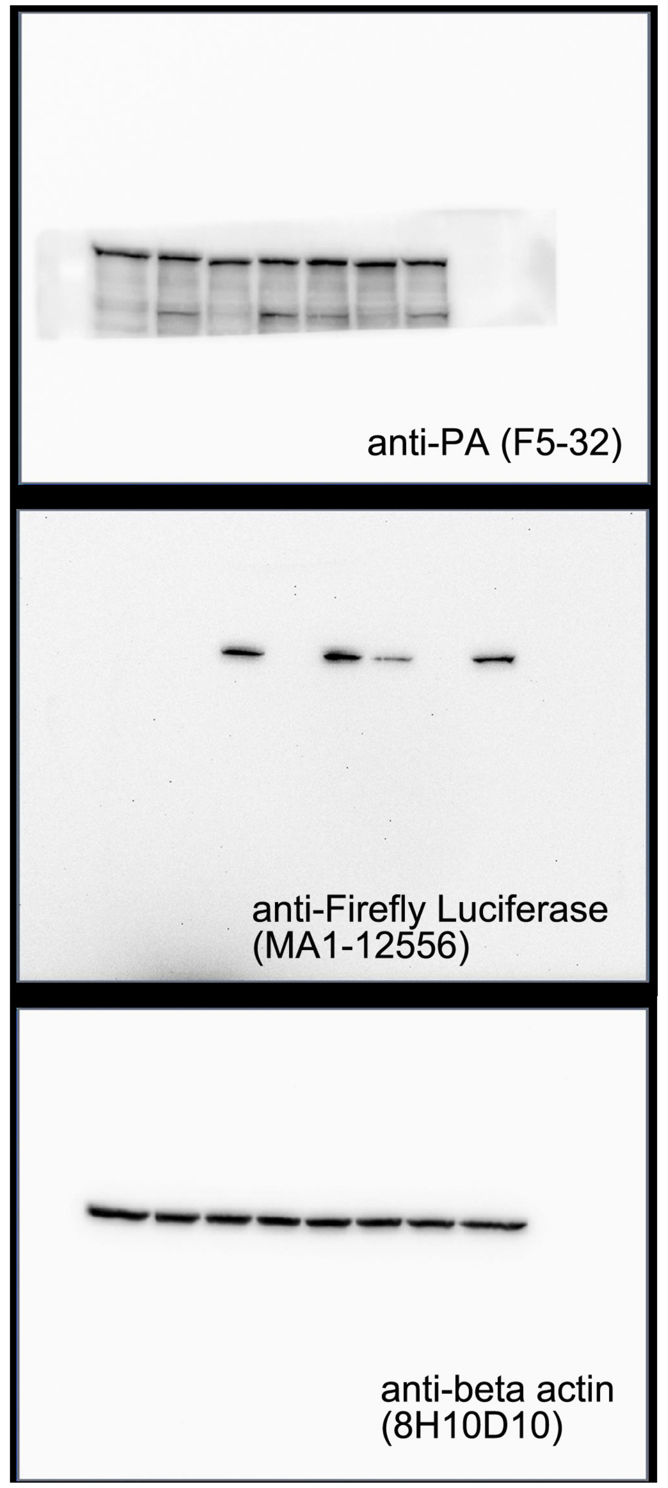

Figure 2f

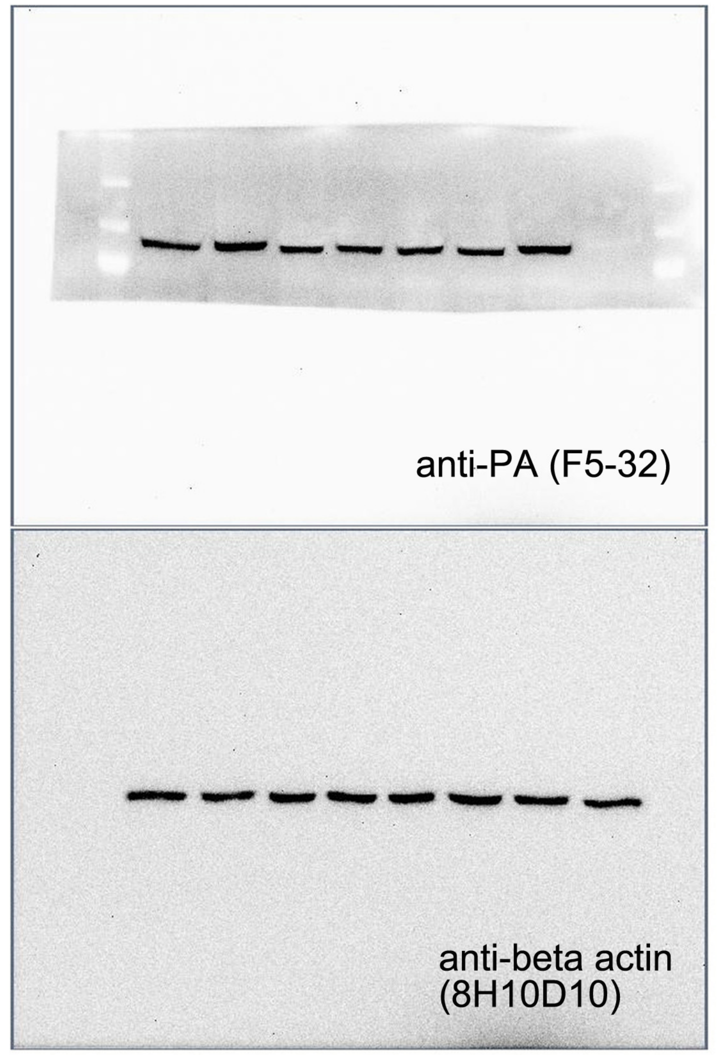

Figure 3h

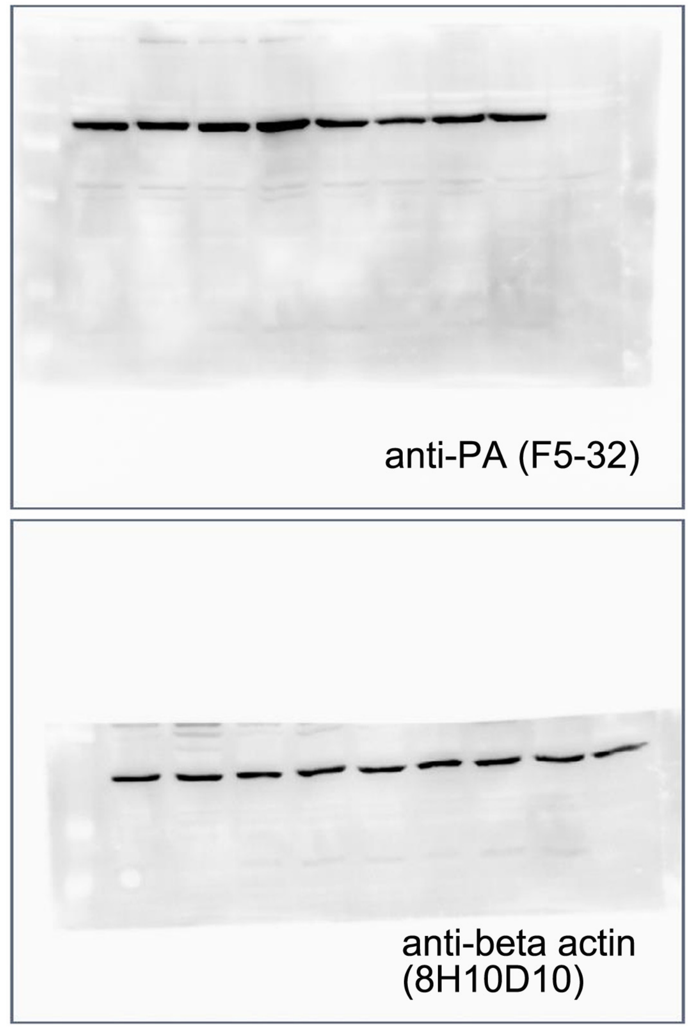

Figure 3i

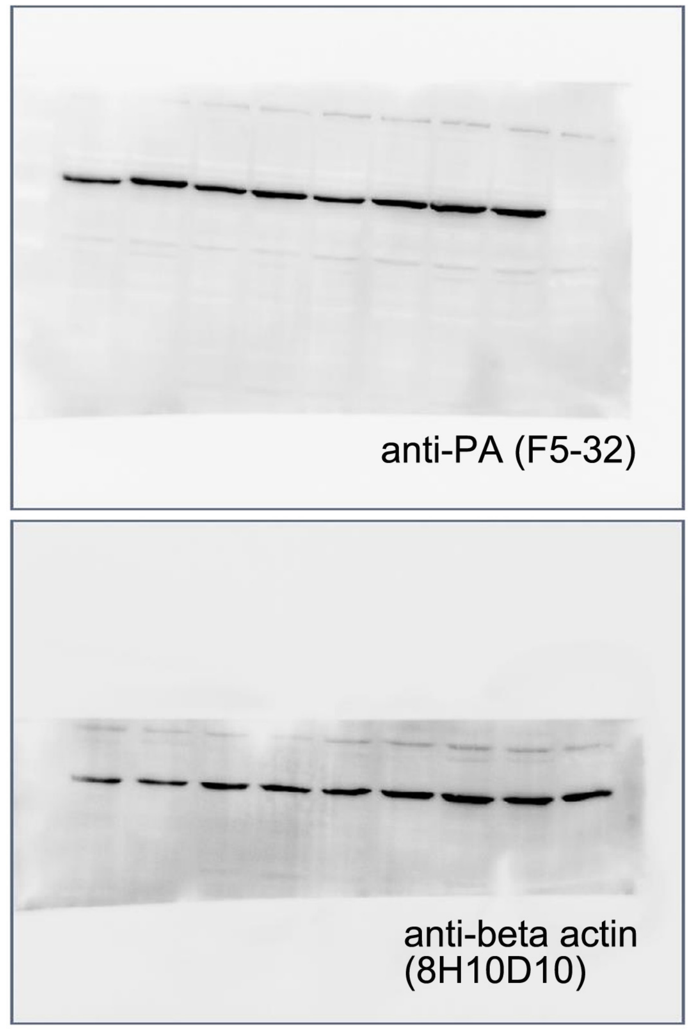

Figure 5c

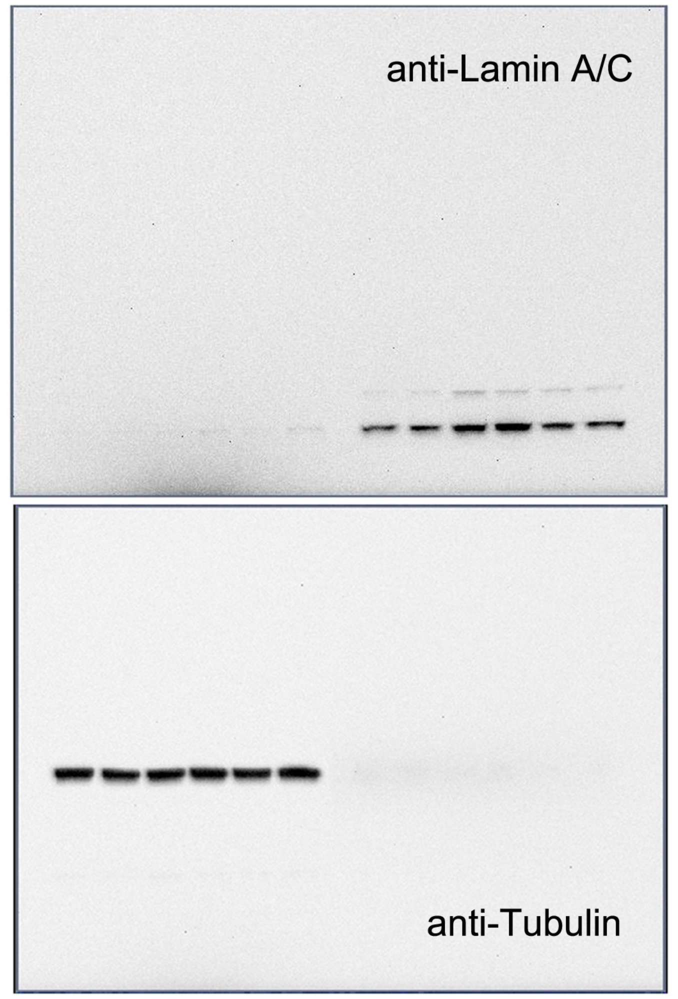

FIgure 7a

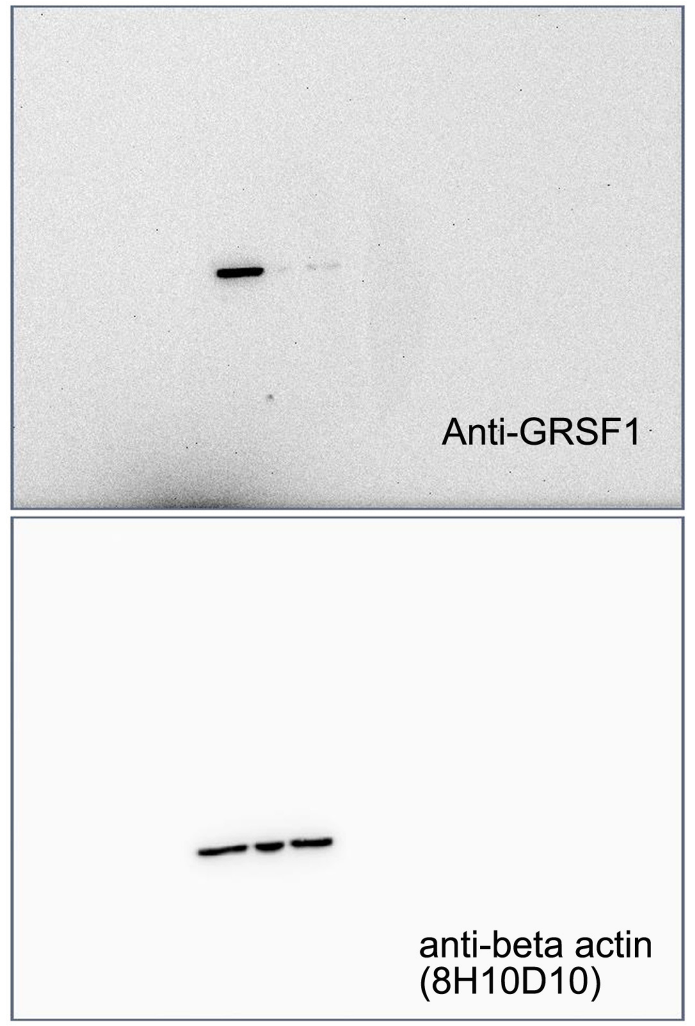

Figure 7d

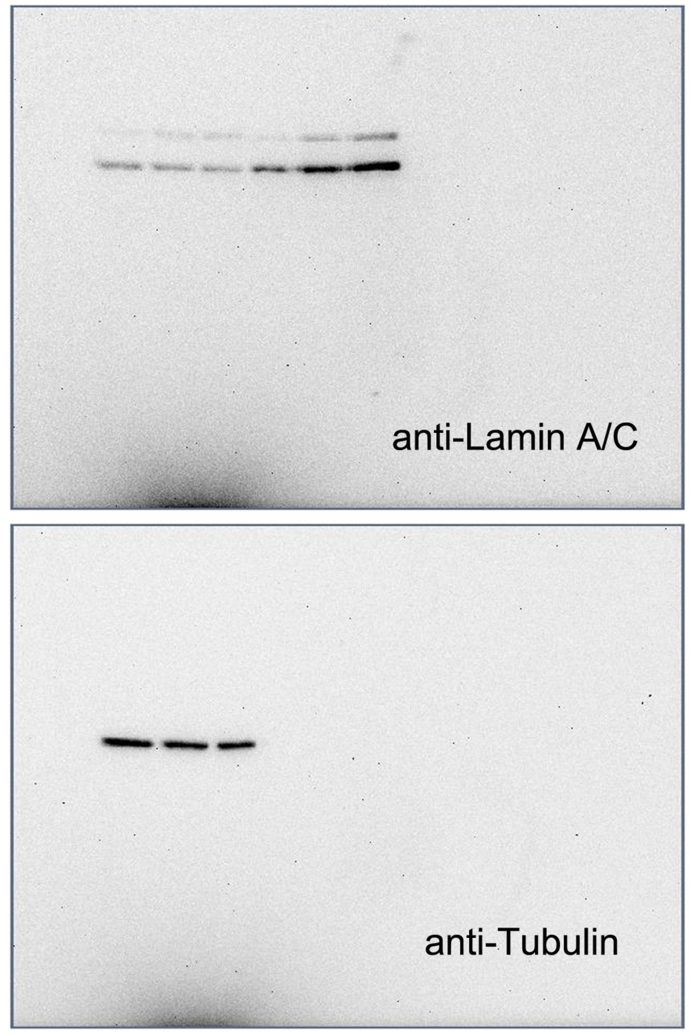

Figure 8c

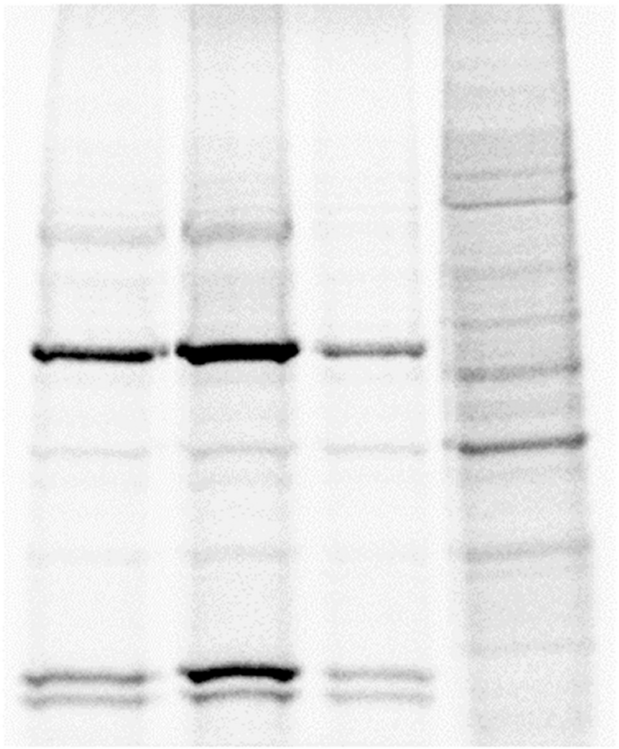

Supplementary Figure 4a

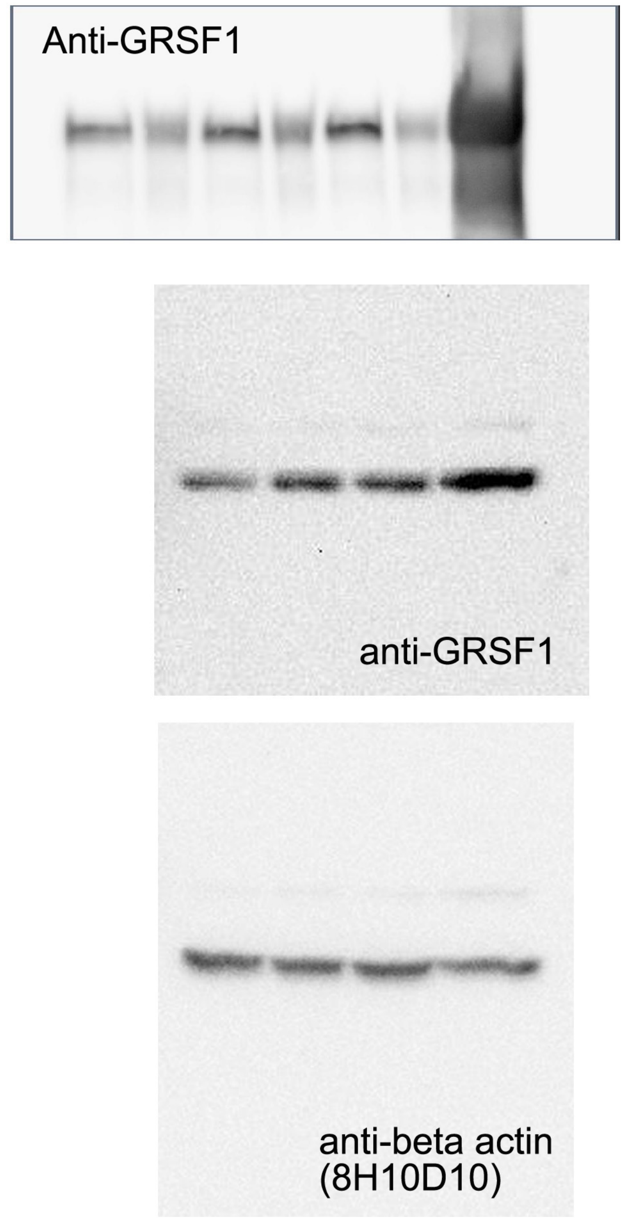

Supplement: Supplementary file 2 — Supplementary Information [file 42003_2022_4082_MOESM2_ESM.pdf]
