# Supplementary material for: Host adaptive mutations in the 2009 H1N1 pandemic influenza A virus PA gene regulate translation efficiency of viral mRNAs via GRSF1
Source: Commun Biol. 2022 Oct 17;5:1102. doi: 10.1038/s42003-022-04082-5 (PMC9576711; doi:10.1038/s42003-022-04082-5)
Supplement: Supplementary file 5 — Reporting summary [file 42003_2022_4082_MOESM5_ESM.pdf]

nature portfolio

Corresponding author(s): Toru Takimoto

Last updated by author(s): Sep 21, 2022

Reporting Summary

Nature Portfolio wishes to improve the reproducibility of the work that we publish. This form provides structure for consistency and transparency in reporting. For further information on Nature Portfolio policies, see our Editorial Policies and the Editorial Policy Checklist.

Statistics

For all statistical analyses, confirm that the following items are present in the figure legend, table legend, main text, or Methods section.

n/a	Confirmed	
☐	☒	The exact sample size (*n*) for each experimental group/condition, given as a discrete number and unit of measurement
☐	☒	A statement on whether measurements were taken from distinct samples or whether the same sample was measured repeatedly
☐	☒	The statistical test(s) used AND whether they are one- or two-sided<br>*Only common tests should be described solely by name; describe more complex techniques in the Methods section.*
☒	☐	A description of all covariates tested
☐	☒	A description of any assumptions or corrections, such as tests of normality and adjustment for multiple comparisons
☐	☒	A full description of the statistical parameters including central tendency (e.g. means) or other basic estimates (e.g. regression coefficient) AND variation (e.g. standard deviation) or associated estimates of uncertainty (e.g. confidence intervals)
☒	☐	For null hypothesis testing, the test statistic (e.g. F, t, r) with confidence intervals, effect sizes, degrees of freedom and P value noted<br>*Give P values as exact values whenever suitable.*
☒	☐	For Bayesian analysis, information on the choice of priors and Markov chain Monte Carlo settings
☒	☐	For hierarchical and complex designs, identification of the appropriate level for tests and full reporting of outcomes
☒	☐	Estimates of effect sizes (e.g. Cohen's *d*, Pearson's *r*), indicating how they were calculated

Our web collection on statistics for biologists contains articles on many of the points above.

Software and code

Policy information about availability of computer code

Data collection	Images for radioactive gels were collected and analyzed using a Biorad Personal Molecular Imager and Quantity One software. Images for Western blots and were collected and analyzed using a Biorad ChemiDoc XRS and Quantity One software. Luciferase assay data was collected and analyzed using a Beckman Coulter DTX 880 Multimode Detector and Multimode Analysis software. Quantitative PCR data was collected and analyzed using an Applied Biosystems 7300 Realtime PCR System and 7300 System software. RNA absorbances for polysome fractionation were collected using a Brandel BR-188 Density Gradient Fractionation System and analyzed with PeakChart software.
Data analysis	Statistical analyses were preformed using GraphPad Prism 9.0 software.

For manuscripts utilizing custom algorithms or software that are central to the research but not yet described in published literature, software must be made available to editors and reviewers. We strongly encourage code deposition in a community repository (e.g. GitHub). See the Nature Portfolio guidelines for submitting code & software for further information.

Data

Policy information about availability of data

All manuscripts must include a data availability statement. This statement should provide the following information, where applicable:

- Accession codes, unique identifiers, or web links for publicly available datasets
- A description of any restrictions on data availability
- For clinical datasets or third party data, please ensure that the statement adheres to our policy

The authors declare that the data supporting the findings of this study are available from the corresponding author upon request. Raw data and gel images for the figures and graphs are provided in supplemental data 1.

Human research participants

Policy information about studies involving human research participants and Sex and Gender in Research.

Reporting on sex and gender	N/A
Population characteristics	N/A
Recruitment	N/A
Ethics oversight	N/A

Note that full information on the approval of the study protocol must also be provided in the manuscript.

Field-specific reporting

Please select the one below that is the best fit for your research. If you are not sure, read the appropriate sections before making your selection.

☒ Life sciences ☐ Behavioural & social sciences ☐ Ecological, evolutionary & environmental sciences

For a reference copy of the document with all sections, see nature.com/documents/nr-reporting-summary-flat.pdf

Life sciences study design

All studies must disclose on these points even when the disclosure is negative.

Sample size	No statistical methods were used to predetermine sample size, and sample size calculations were not preformed.
Data exclusions	No data were excluded from analyses.
Replication	All experiments were repeated with a minimum of 3 biological replicates (independent experiments).
Randomization	Samples were numbered during collection and analyses and identities of samples were only added after data analysis was preformed.
Blinding	No blinding was preformed.

Reporting for specific materials, systems and methods

We require information from authors about some types of materials, experimental systems and methods used in many studies. Here, indicate whether each material, system or method listed is relevant to your study. If you are not sure if a list item applies to your research, read the appropriate section before selecting a response.

Materials & experimental systems

n/a	Involved in the study
☐	☒ Antibodies
☐	☒ Eukaryotic cell lines
☒	☐ Palaeontology and archaeology
☒	☐ Animals and other organisms
☒	☐ Clinical data
☒	☐ Dual use research of concern

Methods

n/a	Involved in the study
☒	☐ ChIP-seq
☒	☐ Flow cytometry
☒	☐ MRI-based neuroimaging

Antibodies

Antibodies used

The following commercially available antibodies were used: Mouse anti-firefly luciferase (MA1-12556, Invitrogen), mouse anti-beta actin (8H10D10, Cell Signaling Technology), rabbit anti-mouse IgG HRP-linked (7076, Cell Signaling Technology), goat anti-mouse IgG1 HRP-linked (ab97240, abcam), Rabbit anti Lamin A/C antibody (#2032, Cell Signaling Technology), Rabbit anti α-Tubulin antibody (#2144, Cell Signaling Technology), goat anti-rabbit IgG HRP-linked (7074, Cell Signaling Technology), rabbit anti-GRSF1 (A305-136A, Bethyl Laboratories), rabbit IgG isotype control (02-6102, Invitrogen), rabbit anti-GRSF1 (PA5-116809, Invitrogen).

Additionally, for detection of PA, mouse anti-PA (Clone F5-32) was used (DOI:10.1016/j.virol.2012.01.015).

Validation

All commercially available antibodies have validation data provided on the suppliers website. Validation for mouse anti-PA F5-32 can be found in DOI:10.1016/j.virol.2012.01.015.

Eukaryotic cell lines

Policy information about cell lines and Sex and Gender in Research

Cell line source(s)

293T cells (CRL-3216), A549 cells (CCL-185), MDCK cells (CCL-34), and Calu-3 cells (HTB-55) were obtained from ATCC.

Authentication

All cell lines were not authenticated.

Mycoplasma contamination

All cell lines tested negative for mycoplasma contamination.

Commonly misidentified lines
(See ICLAC register)

No commonly misidentified cell lines were used.
